# Exercise-Induced Transcriptional Changes in Aged Skeletal Muscle

**DOI:** 10.1093/geroni/igab046.2549

**Published:** 2021-12-17

**Authors:** Yori Endo, Shayan Olumi, Mehran Karvar, Indranil Sinha, Yuteng Zhang

**Affiliations:** 1 Harvard Medical School, Boston, Massachusetts, United States; 2 Northwestern University, Evanston, Illinois, United States; 3 Brigham and Women's Hospital, Harvard Medical School, Boston, Massachusetts, United States; 4 Birgham and Women's Hospital, Harvard Medical School, Boston, Massachusetts, United States

## Abstract

Exercise is beneficial for physical functions across all ages. However, the response to exercise shifts from anabolism, resulting in limited gain of muscle strength and endurance. These changes likely reflect age-related alterations in transcriptional response underlying the muscular adaptation to exercise. The exact changes in gene expression accompanying exercise, however, are largely unknown, and elucidating them is of a great clinical interest for optimizing the exercise-based therapies for sarcopenia. In order to characterize the exercise-induced transcriptomic changes in aged muscle, a paired-end RNA sequencing was performed on the rRNA-depleted total RNA extracted from the gastrocnemius muscles of 24 months-old mice after 8 weeks of regimented exercise (exercise group) or sedentary activities (sedentary group). Differential gene expression analysis revealed upregulations in the group of genes involved in neurotransmission. In particular, genes encoding the transporters and receptor components of glutaminergic transmission were significantly upregulated in exercised muscles, as exemplified by Gria 1, Gria 2 and Grin2c encoding glutamate receptor 1, 2 and 2C respectively, Grin1 and Grin2b encoding N-methyl-D-aspartate receptors (NMDARs), Nptx1 responsible for glutaminergic receptor clustering, and Slc1a2 and Slc17a7 regulating synaptic uptake of glutamate. These changes were accompanied by an increase in post-synaptic NMDARs and acetylcholine receptors (AChRs), as well as their innervation at neuromuscular junctions (NMJs). These results suggest that neural responses predominate aged skeletal muscle following exercise, and indicate a possibility that glutaminergic transmission at NMJs may be responsible for synaptic protection and neural remodeling accompanying the exercise-induced functional enhancement in aged skeletal muscle.

